# Polymorphism of Genes Encoding Inflammatory Interleukins and the Risk of Anterior Cruciate Ligament Injury: A Systematic Review and Meta-Analysis

**DOI:** 10.3390/ijms25094976

**Published:** 2024-05-02

**Authors:** Katarzyna Lorenz, Andrzej Mastalerz, Anna Cywińska, Aleksandra Garbacz, Ewelina Maculewicz

**Affiliations:** 1 Faculty of Physical Education, Jozef Pilsudski University of Physical Education, 00-968 Warsaw, Poland; katarzyna.lorenz@awf.edu.pl (K.L.); andrzej.mastalerz@awf.edu.pl (A.M.); 2 Faculty of Biological and Veterinary Sciences, Nicolaus Copernicus University in Torun, 87-100 Torun, Poland; anna_cywinska@weterynaria.pl; 3 Faculty of Animal Genetics and Conservation, Warsaw University of Life Sciences, 02-786 Warsaw, Poland; aleksandra_garbacz1@sggw.edu.pl; 4 Department of Laboratory Diagnostics, Military Institute of Aviation Medicine, 01-755 Warsaw, Poland

**Keywords:** interleukin, gene polymorphism, anterior crucial ligament, injury, soft tissue

## Abstract

Sport injuries, including the anterior crucial ligament rupture (ACLR) seem to be related to complex genetic backgrounds, including the genes responsible for inflammatory response. This review and meta-analysis investigated the contribution of the polymorphisms of genes encoding inflammatory cytokines and their receptors to the risk of ACLR. The scientific databases Science Direct, EBSCO host, Scopus, PubMed, and Google Scholar were screened (completed on 14 June 2023) according to the established inclusion/exclusion criteria (only fully accessible, original, human case–control studies written in English concerning the effect of interleukin genes’ polymorphisms on the occurrence of ACL injury were included) and statistical meta-analysis using R version 4.0.3 was performed. The PRISMA methodology was used to review articles. The review protocol was registered under the number CRD42024514316 in the Prospero database. Eighty-nine studies were identified and narrowed down to three original case–control studies used for the meta-analysis. The studies analyzed Polish, South African, and Swedish cohorts, altogether 1282 participants. The candidate polymorphisms indicated in the studies involved IL6 rs1800795, IL6R rs2228145 and IL1B rs16944. The systematic review showed the relationships between IL6 rs1800795 polymorphism and ACLR in the Polish subpopulation, and IL6R rs2228145 and IL1B rs16944 in the South African subpopulations. The meta-analysis revealed that the IL6 rs1800795 CG genotype was over-represented (OR = 1.30, 95% CI 1.02–1.66), while the CC genotype was under-represented (OR = 0.75, 95% CI 0.54–1.03) in ACLR subjects, but no significant impact of IL6R rs2228145 was shown. Additionally, a tendency of the IL1B rs16944 CT genotype to be protective (OR 0.89, 95% CI 0.70–1.14), while the TT to be a risk genotype (OR 1.19, 95% CI 0.84–1.68) was observed. Thus, the relationship between the interleukin receptor IL6R rs2228145 and ACLR risk was not confirmed. However, the impact of genes coding pleiotropic IL6 rs1800795 on the incidences of ACLR was clear and the effect of pro-inflammatory IL1B rs16944 was possible.

## 1. Introduction

Physical activity brings many health benefits and systemic positive effects on the human body [1]. However, improperly designed or excessive physical effort increases the risk of injury in the musculoskeletal system [2]. Physically active people, both professional and amateur athletes, are particularly vulnerable to injuries, mainly involving the soft tissues including the muscles, ligaments, and tendons of the limbs [2,3,4]. Injuries most frequently affect the ankle and knee joints [3,4], with the anterior cruciate ligament rupture (ACLR) being one of the most common injuries in sport as its incidence has doubled over the last twenty years [5]. This type of injury is diagnosed more frequently in men due to the fact that they participate in contact sports more frequently; however, the risk of ACL injury is estimated to be two to eight times greater in women [5]. About 75% of these injuries occur in the non-contact activities, i.e., a sudden change of speed and direction without contact with other players, even in high-collision sports, which further suggests that genetic background increases the risk of injury [6,7]. Hypotheses regarding the relationship between genetics and the incidences of injuries appeared even before the era of genetic research. In a study by Flynn et al. [8], a familial predisposition was indicated based on the observation that patients with an ACL injury were twice as likely to have relatives with a similar injury than participants without this type of injury. The association between genetic variants and sport-related traits is a relatively new area of research. Due to the ongoing trends of maintaining a healthy lifestyle, as well as the increasing efforts of sportsmen playing at a highly professional level, it is crucial to widely understand all of the possible genetic components that may influence an athlete’s performance and their risk of injury [9].

Previous studies have confirmed the impact of gene polymorphisms on the incidences of spontaneous and non-contact injuries. One of the first candidates analyzed in the context of soft-tissue injuries were the genes encoding collagen proteins. Many researchers have proved the influence of certain genotypes in collagen gene polymorphisms on the occurrence of soft-tissue injuries [10]. The role of interleukins and their genes in sport and injuries received less attention. However, soft-tissue injury is a complex process that includes inflammation and remodeling, which are mediated by interleukins. Thus, additionally examining the genes encoding interleukins may help achieve a wider understanding of the injury process and risk. Doubtlessly, injuries often result from accidents; however, they also occur spontaneously or as a consequence of fatigue. Repeated submaximal loading leads to microinjuries in the structural extracellular matrix (ECM) and the structurally stable connective tissue elements such as ligaments and tendons [11]. This microdamage is somehow beneficial, as it leads to adaptation to the increasing workload by muscle hypertrophy and increasing the stiffness of connective tissue, but proper cell signaling is crucial for these effects [12,13]. On the other hand, microinjuries occurring as a result of participating in professional sporting competitions, but also training sessions, may reduce blood flow and oxygen supply and promote collagen degeneration. Thus, a proper amount of time for healing (reflected by the rest time recommended in all kinds of training) and cell signaling is crucial. Accumulated fatigue damage from microtrauma without a sufficient healing period may progress to complete failure, which is a common element of the pathogenesis of ACLR [7,14].

Microinjuries trigger the subclinical inflammatory response, mediated by wide variety of cytokines. Cytokines are cellular signaling molecules involving interleukins, interferons, chemokines, growth factors, tumor necrosis factors family, and adipokines [13,15]. About 200 cytokines have been identified to date, including many pro- and anti-inflammatory ones, which are also important in the body’s response to exercise [13,16].

Interleukin 1 (IL-1) is a highly pro-inflammatory cytokine often examined in combination with another pro-inflammatory cytokine, tumor necrosis factor (TNF). The IL-1 family consists of 11 members, 7 proinflammatory and 4 with antagonistic or anti-inflammatory properties, regulating both acute and chronic inflammatory states [17,18,19,20]. IL-1α and IL-1β are expelled from the cells early in the course of inflammation, resulting in localized and then systemic inflammation [17,20]. IL-1 can activate and enhance defense mechanisms, although the line between clinical benefit and harm is very narrow, so the elevated levels do not necessarily contribute to regenerative processes [18]. IL-1 regulates the proliferation of fibroblasts and synoviocytes of the synovial membrane in the joints, the secretion of collagenase, as well as catabolism in the muscles. Knee ligament injuries are accompanied by an increased production of interleukin-1β (IL-1β), encoded by the *IL1B* gene [21]. IL-1β is a strong pro-inflammatory cytokine, produced at the site of an injury, mainly by macrophages. It enhances the inflammatory response by proinflammatory cell signaling, including the stimulation of the production of other cytokines, e.g., interleukin-6 [18,19]. The IL-1 receptor antagonist (IL-1Ra) acts as an inhibitor of IL-1α and IL-1β activity [19,22]. The balance between agonistic and antagonistic members of the IL-1 family is crucial for the inflammatory process and the role of IL-1β and IL-1Ra in the context of knee injury has been discussed most frequently [21,23].

Interleukin 6 (IL-6) is a pleiotropic cytokine involved in inflammatory response as well as the regulation of metabolic and regenerative processes [13,24,25]. It exerts biological effects by binding to the interleukin-6 receptor (IL-6R), encoded by the *IL6R* gene. IL-6 is frequently discussed in the context of exercise and adaptation as its concentration in plasma depends on the duration and intensity of exercise, as well as the involved muscle mass [13,26]. Despite its adaptational metabolic effect, IL-6 has various biological properties such as stimulating the liver to the production of acute-phase proteins in the onset of an acute-phase response, as well as the production of antibodies [13,27,28]. After the injury of tendons and ligaments, IL-6 is secreted by fibroblasts and is involved in the regulation of defense mechanisms including the immune response, inflammatory reaction, and hematopoietic processes [29]. In the muscles, the expression of mRNA for IL-6 increases after injury, as IL-6 regulates myoblast proliferation and differentiation, though it is postulated that IL-6 may be active in the regeneration of tendons [30]. IL-6 has both pro- and anti-inflammatory activities [13] and can even promote the progression from acute into chronic inflammation [29]. Its levels in the synovial fluid in the knee are the highest 2–3 days after injury, suggesting its role in the early phase of tissue response to ACLR [21,31]. When IL-6 concentrations are pathologically high, TGF-β mediates the immunosuppression, acting as a counterpart of IL-6 [32]. Synergy has been observed between IL-6 and IL-17 in inhibiting cellular apoptosis; therefore, promoting the development of chronic inflammation, tumor growth, viral persistence, as well as autoimmune diseases [33]. IL-6 plays a key role in bone resorption and cell apoptosis and is involved in collagen synthesis [34]. In the context of knee ligament injuries, it should be mentioned that in vitro studies have shown that cyclic stretching of human tendon fibroblasts increased IL-6 secretion and a similar phenomenon may also be expected in pathological conditions of tendons or ligaments [35].

Cytokines have been widely investigated in numerous fields; however, the genetic background related to cytokine response in sport injuries and sport-related traits has received only a little attention. A study by Burger et al. [36] has shown that the genotype AA of *IL6R* polymorphism rs2228145 is associated with a lower risk of carpal tunnel syndrome in the South African population. Dakin et al. [37] revealed that CXCL8 expression was increased after Achilles tendon rupture. It was also proven that the interaction between *COL5A1* BstUI and *IL1B -31T>C, -511C>T*, *IL6 -172G>C*, and *IL1RN* VNTR polymorphisms increased the risk of Achilles tendinopathy [38]. A study by Nakama et al. [39] has indicated that the levels of IL6 mRNA were increased in the subacromial synovium and in ruptured rotator cuff tendons. The development of molecular research techniques provides more opportunities to analyze genetic variability and combine this with the properties of encoded proteins, as well as with the functional properties of tissues. Studies concerning the involvement of genes encoding interleukins in the onset of inflammation pose a new direction in genetic research on damage to soft tissue in the musculoskeletal system. Cytokines, due to their roles in modulating inflammatory response and tissue remodeling, are important in the onset of injuries, and the genetic background related to cytokines can be considered an additional marker for the risk and the onset of sport-related soft-tissue damage.

Therefore, the aim of this review and meta-analysis was to investigate the relationship between the single-nucleotide polymorphisms (SNPs) of genes encoding inflammatory interleukins IL-1, IL-6, and their receptors and the risk of anterior cruciate ligament rupture.

## 2. Materials and Methods

### 2.1. Selection of the Studies

**Figure 1:**
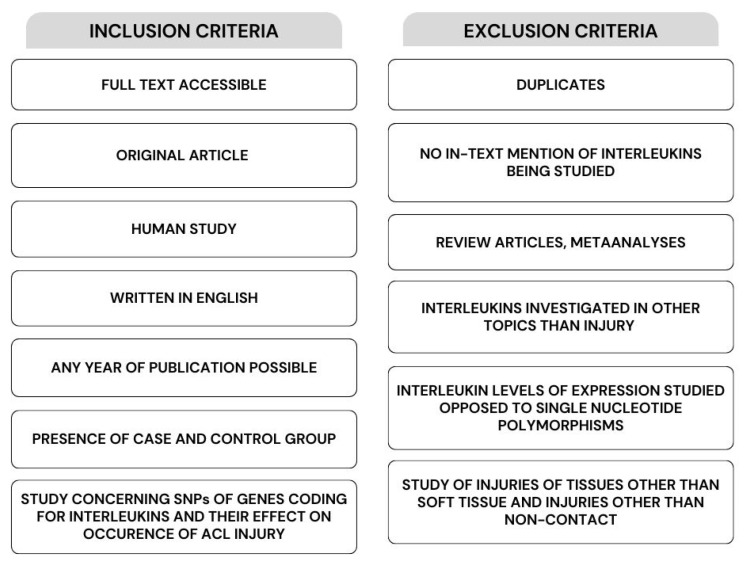
Inclusion and exclusion criteria for meta-analysis.

The articles for this study were selected by searching the databases: Science Direct, Scopus, EBSCO host, PubMed, and Google Scholar. The search was completed on 14 June 2023. The following keywords were used: injury, gene, interleukin, sport, ACL, connected by the: AND, OR. This method revealed 158,060 articles, of which 5566 were eliminated because of duplication. Additionally, 152,405 articles were excluded due to abstracts and titles not being relevant to the studied topic, the publication type (systematic reviews and meta-analyses), and non-human studies. Ultimately, 89 publications were assessed for eligibility, which resulted in the inclusion of three articles for analysis. Further inclusion and exclusion criteria are presented in Figure 1. The review was performed according to the guidelines of the Preferred Reporting Items for Systematic Review and Meta-Analysis (PRISMA). The review protocol was registered under the number CRD42024514316 in the Prospero database.

**Figure 2:**
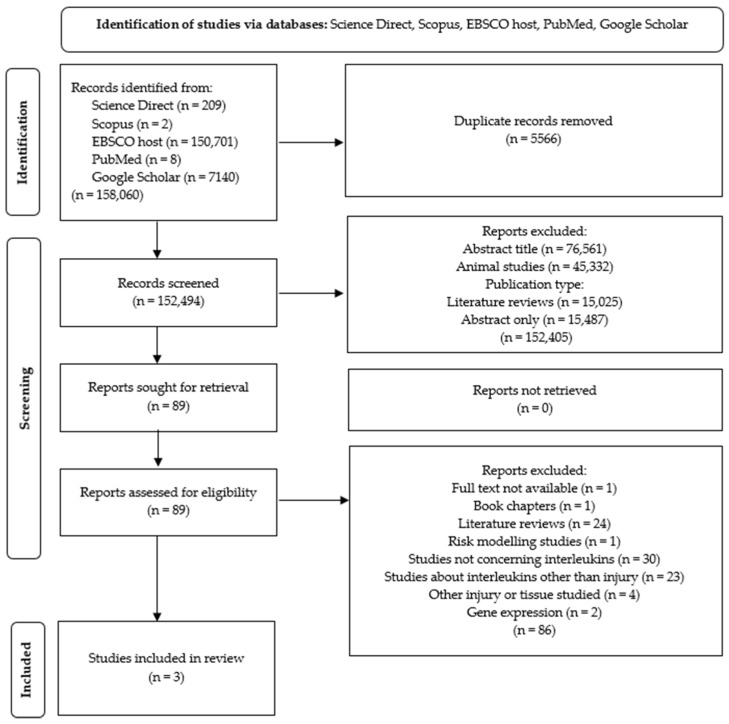
Study selection flow chart (PRISMA flow diagram for systematic reviews).

Firstly, the articles were screened by titles and abstracts to check if interleukins were mentioned. If the abstract did not mention interleukins but contained detailed information about the methods used, this was a premise to exclude the article from further inspection. However, if interleukins were not mentioned in the abstract while the description of the investigated genes and parameters was not described in detail, the whole text was screened. Records were excluded from the final analysis if one of the following criteria was fulfilled: no full-text access, non-human studies, studies not concerning interleukins, systematic reviews, meta-analyses, books, studies on topics other than injuries of the ligaments, gene expression instead of genetic variability, and risk modelling. To be included in this review and meta-analysis, articles had to meet all of the individual inclusion criteria and none of the exclusion criteria. The selection process was conducted by one investigator, who screened each record by reading the title, abstract, as well as the whole article. Data collection was also performed by the same investigator who analyzed the selected articles. The algorithm showing the consecutive steps in the study selection process is shown as a flow diagram (Figure 2).

### 2.2. Quality Assessment of the Selected Articles

Data from the studies selected for inclusion for the review and meta-analysis were extracted and synthesized in Table 1. Quality assessment of the selected studies was performed based on the *Strengthening the Reporting of Mendelian Randomization Studies* guidelines (STROBE-MR). The range of quality assessment scores were as follows: >85% meant a low risk of bias, 75–85% a medium risk, and <75% a high risk. All of the selected articles were assessed individually and received scores between 0 and 1 for each criterion (title and abstract, background, objectives, study design and data sources, assumptions, statistical methods, descriptive data, main results (results), key results (discussion), limitations, interpretation, generalizability, and other information). All of the articles selected for further analysis received scores above 85%, which meant a low risk of bias.

### 2.3. Statistical Meta-Analysis

R software (version 4.0.3, R Foundation for Statistics Computing, https://cran.r-project.org (accessed on 1 July 2023)) was used to perform all of the statistical analyses. The overall odds ratio (OR) from all data was calculated using the Mantel–Haenszel method in one analysis. The heterogeneity of the groups was tested to check if a meta-analysis could be performed. In order to check this criterion, the Woolf test was used and the meta function from the meta.bin package was used to perform the calculations. The Baujat graph (created using the baujat function) and a funnel plot (funel function in the meta package) were used for sensitivity analysis to detect different studies affecting the lack of group homogeneity. The meta package forest.plot function was also used to make forest plots.

The probability of the null hypothesis stating that there is no heterogeneity between the studies as opposed to significant heterogeneity between the studies is shown on the forest plots as the Chi-squared test *p*-value. Due to the high heterogeneity of the groups (I2 > 40% and *p*-value < 0.10), a random effects model was selected. When I2 was less than 40% and the *p*-value greater than 0.10, a fixed-effect model was chosen. Due to the fact that all of the articles taken into account used the odds ratio (OR), it was chosen as a measure of the effect under consideration.

## 3. Results

### 3.1. Selection of the Studies

The initial screening revealed 158,060 works found through the scientific databases search, of which 157,971 were excluded due to duplication, abstracts and titles, publication type, or irrelevant study design, with 89 works remained for further screening. The articles included in the final meta-analysis, selected according to the inclusion/exclusion criteria, were fully accessible case–control original articles written in English, dealing with the relationships between genes encoding interleukins and anterior cruciate ligament ruptures in humans. During the study selection process, 86 out of 89 works were removed and three original articles remained for further analysis. The selected studies examined Polish, Swedish, and South African subpopulations. All of them analyzed females and males and also differentiated a subgroup of participants who suffered from a non-contact ACLR, which is crucial to investigating the impact of genetic background on the incidence of injury. All of the studies analyzed the following polymorphisms: *IL1B* rs16944, *IL6* rs1800795, and *IL6R* rs2228145.

The studies indicated that the *IL6* rs1800795 polymorphism was significantly related to ACLR incidences; however, no significant differences were found for *IL6R* rs2228145 and *IL1B* rs16944 between the control group and the ACLR group for the Polish cohort [40]. For the South African group, the *IL6R* rs2228145 CC genotype was significantly overrepresented in the control group in comparison with the ACLR group [41]. The TT genotype of the *IL1B* rs16944 polymorphism was underrepresented in the control group compared to the non-contact ACLR group for female South African participants [42]. The details concerning the selected studies and their main findings are presented in Table 1.

### 3.2. Description of the Selected Studies

The meta-analysis involved three articles by Lulińska-Kuklik et al. [40], by Suijkerbuijk et al. [41], and by Rahim et al. [42]. The study by Lulińska-Kuklik et al. [40], entitled “Are IL1B, IL6 and IL6R Gene Variants Associated with Anterior Cruciate Ligament Rupture Susceptibility?”, investigated four polymorphisms within the genes encoding interleukins: IL1B rs16944, IL1B rs1143627, IL6 rs1800795, and IL6R rs2228145. The studied cohort consisted of 423 Polish participants (males and females), 229 of them constituted the case group with diagnosed ACLR, qualified for reconstruction surgery, and 194 healthy participants were the control group with no history of ACL injury. All participants in the case group suffered from a non-contact injury and were professional soccer players. The male participants were recruited from the first, second, and third divisions of the Polish soccer league and the female participants were from the first division. No associations were found between the SNP-SNP interactions and ACLR for the dominant, codominant, and recessive models. No significant differences were found for genotypes and allele frequencies between the case and control groups. However, it was found that the IL6 rs1800795 polymorphism was significantly associated with ACLR for the recessive (G/G-C/G vs. C/C OR 1.74, 95% CI 1.08–2.81, *p*-value = 0.032 sex-adjusted), codominant and overdominant (heterozygosity associated with OR 0.56, 95% CI 0.38–0.83, *p*-value 0.007 sex-adjusted) models [40].

The paper by Suijkerbuijk et al. [41], entitled “Functional polymorphisms within the inflammatory pathway regulate expression of extracellular matrix components in a genetic risk dependent model for anterior cruciate ligament injuries”, examined three polymorphisms: IL1B rs16944, IL6 rs1800795, and IL6R rs2228145. Two cohorts of participants from Sweden and South Africa were examined. The case group with ACLR consisted of 79 Swedish participants and 98 South African participants recruited from orthopedic clinics, and the control group involved 116 Swedes and 100 South Africans who were physically active and seemingly healthy. All members of the Swedish case group suffered from a non-contact injury to the ACL. In the South Arican group, 51 patients ruptured their ACL through non-contact injury, while 47 participants experienced a contact injury. The IL6R rs2228145 CC genotype was significantly overrepresented in the control group in comparison to the ACLR group in the South African cohort (*p*-value = 0.028). Among the male participants, the T-C-G of the COL5A1-IL1B-IL6 allele combination, as well as the T-C-A of the COL5A1-IL1B-IL6R allele combination, were significantly underrepresented in the control group in comparison to the non-contact ACLR group for the Swedish cohort (*p* = 0.034, *p* = 0.044, respectively) [41].

The last article included in this meta-analysis was the study by Rahim et al. [42], entitled “Modulators of the extracellular matrix and risk of anterior cruciate ligament ruptures”, which examined three polymorphisms: IL1B rs16944, IL6 rs1800795, and IL6R rs22281457. The case group consisted of 234 South African participants with a diagnosed ACLR, recruited from an orthopedic clinic, out of which 135 suffered a non-contact injury. The control group included 232 South Africans. The participants were primarily recreational athletes. When only females were taken into consideration, the TT genotype of the IL1B rs16944 was significantly underrepresented in the control group compared to the non-contact ACL injury group (OR 3.06, 95% CI 1.09–8.64, *p* = 0.039). In addition, it was found that the AC genotype in the IL6R rs2228145 polymorphism was significantly overrepresented in the control group compared to the non-contact ACL injury group (*p*-value = 0.036) [42].

### 3.3. Meta-Analysis Results

**Figure 3:**
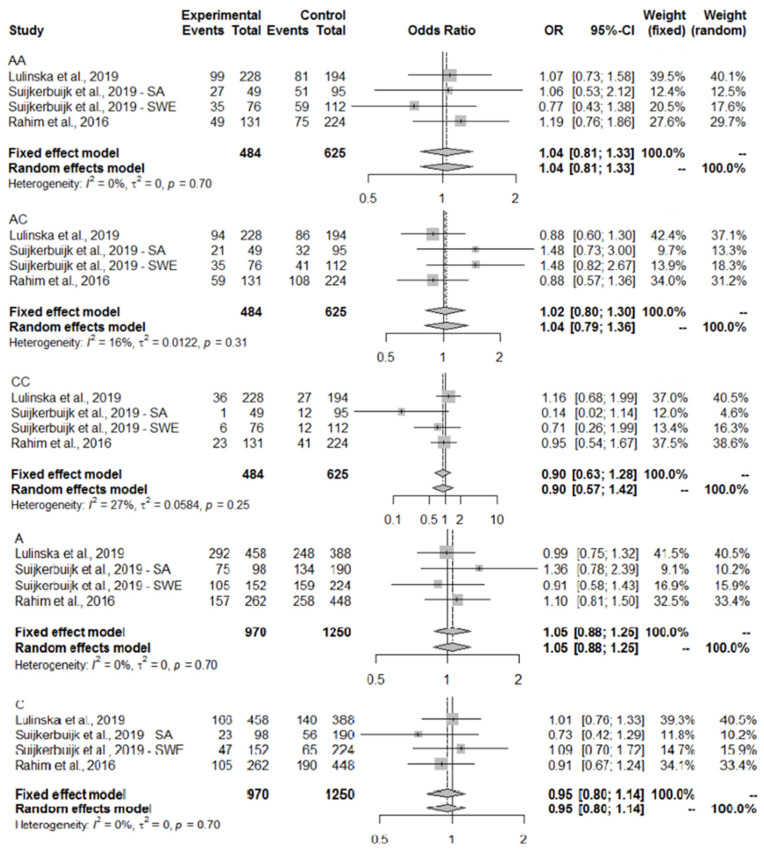
Pooled ORs from three articles on associations between *IL6R* (rs2228145) and ACLR. The size of the grey shaded area indicates the weight of each study and the horizontal lines represent a 95% CI. Abbreviations: AA—adenine-adenine; AC—adenine-cytosine; CC—cytosine-cytosine; A—adenine; C—cytosine.

**Figure 4:**
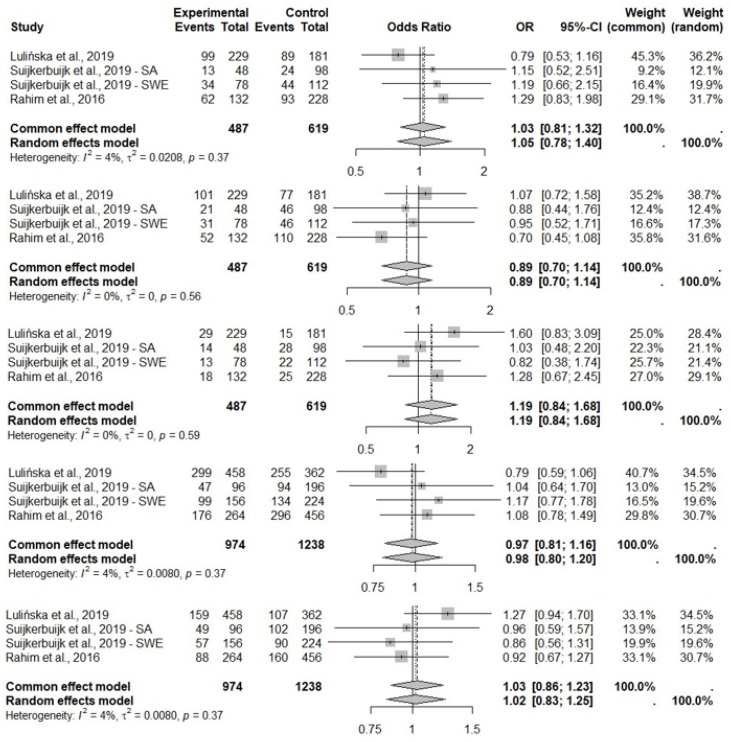
Pooled ORs from the three articles on associations between *IL1B* (rs16944) and ACLR. The size of the grey shaded area indicates the weight of each study and horizontal lines represent a 95% CI.

**Figure 5:**
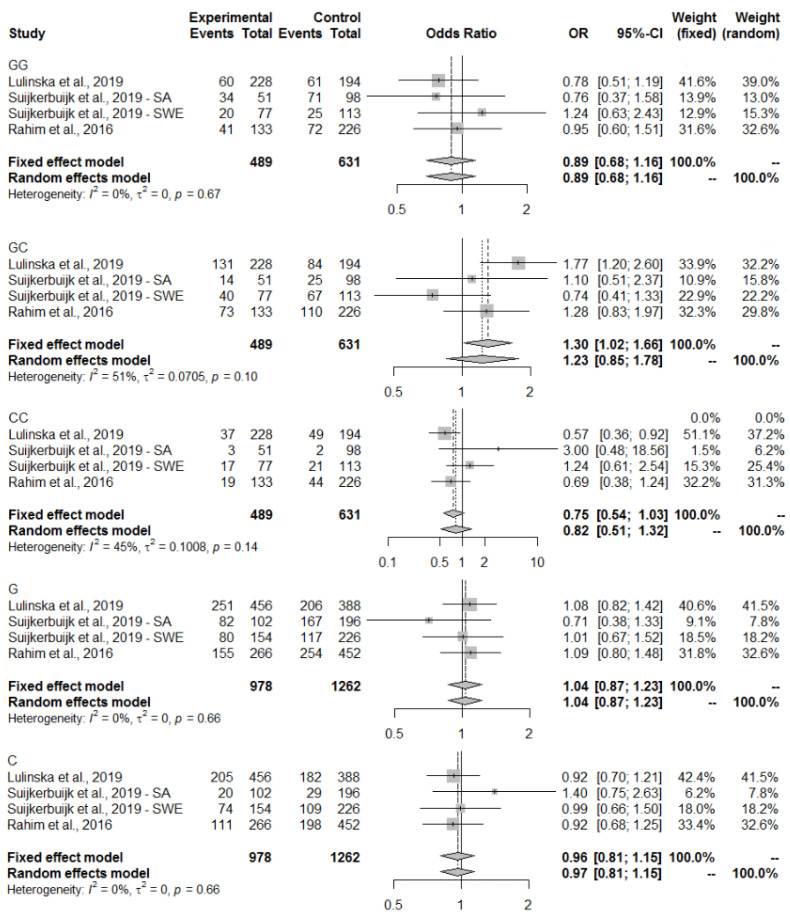
Pooled ORs from the three articles on associations between *IL6* (rs1800795) and ACLR. The size of the grey shaded area indicates the weight of each study and the horizontal lines represent a 95% CI. Abbreviations: GG—guanine-guanine; GC—guanine-cytosine; CC—cytosine-cytosine; G—guanine; C—cytosine.

The meta-analysis of the abovementioned studies on IL6R (rs2228145) genotype in the context of ACLR has shown pooled OR results close to 1, which means that there was no relationship or only a negligible impact (Figure 3). A fixed-effect (common) model was used to analyze the results for all genotypes and alleles, due to I2 < 40% and *p*-value > 0.05. Analyzing the results within IL1B (rs16944), for the CT genotype, a protective-effect bias was shown with a common effect size of OR = 0.89 (95% CI 0.70–1.14), and for the TT genotype a slight tendency indicating an ACLR-risk genotype with OR = 1.19 (95% CI 0.84–1.68), but for both results the confidence interval contains 1, so no firm conclusions can be drawn. The remaining OR results oscillate around 1, indicating no effect of the genotypes or single alleles on the trait under investigation (Figure 4). There was a relationship between IL6 (rs1800795) and ACLR, and the GC genotype was found to be related to ACLR with a pooled OR =1.30 (95% CI 1.02–1.66). A fixed-effect model was chosen because of I2 = 51% and *p*-value = 0.10. The presence of the CC variant was negatively related to ACLR with a pooled OR = 0.75 (95% CI 0.54–1.03) in a fixed-effect model (I2 = 45%, *p*-value = 0.14) (Figure 5). Only non-contact ACL injuries were taken into consideration during this meta-analysis. The Swedish and South African cohorts [41] were examined separately due to the significant differences between the genotype and allele frequency distributions between the two groups. The meta-analysis results for the three common polymorphisms studied in the above-mentioned three selected articles (IL1B rs16944, IL6 rs1800795, and IL6R rs2228145) are presented in Figure 3, Figure 4 and Figure 5.

## 4. Discussion

The aim of this systematic review and meta-analysis was to summarize the findings regarding the relations between genetic variability and the polymorphisms of genes encoding pro-inflammatory *IL1B*, pleiotropic *IL6*, and its receptor *IL6R* and incidences of ACLR. This topic is important due to the fact that the cytokine-mediated regulation of inflammatory response and tissue remodeling is crucial for both the risk of injury and further healing and the outcome of the injury. The mechanisms are similar for professional athletes participating in training sessions, contests, and sporting events, and for amateurs who wish to maintain a healthy lifestyle involving physical activity at moderate levels.

The result of database screening by selected keywords indicated 89 records. The vast majority of these were excluded as they dealt with injuries to structures other than the ACL, including medical conditions such as carpal tunnel syndrome, rotator cuff tendon injury, Achilles rupture and tendinopathy, cardiovascular diseases, preterm, prelabor, and birth, abdominal aortic aneurysm, myocardial infraction, squamous cell carcinoma psoriasis, artery atherosclerotic stroke, neonatal morbidity, or type II diabetes mellitus. Some studies not included in this systematic review and meta-analysis examined other genes, such as *MMP3*, *TNFα*, *ACTN3*, *COL1A1,* or the *PPAR* gene family. Although these are of major importance for a genetic predisposition to ACLR, they are not strictly related to the onset of inflammation and healing. Other works have analyzed gene expression and the concentration of cytokines, which additionally confirm the role of cytokines but did not examine genetic background. A few reviews were focused on genes associated with sporting performance; however, they did not cover injuries and if they did, they discussed other genes. This systematic review shows that the studies concerning the influence of polymorphisms within genes encoding interleukins and the risk of soft-tissue injury are still scarce, as there were three studies left for further investigation after selection.

The results of the meta-analysis did not confirm the unequivocal role of all candidate polymorphisms examined in the included studies [40,41,42]. Only the polymorphisms of *IL6* rs1800795 were confirmed by meta-analysis to be significantly related to ACLR, with GC genotype indicating an increased risk of ACLR and the CC genotype being protective. The studies included in the meta-analysis, taken individually, present different trends; thus, this may have suggested that gender and nationality should be considered [10]. In the Polish subpopulation, *IL6* rs1800795 was significantly related to incidences of ACLR [40]; in Swedish males, but not females, a haplotype *COL5A1*-*IL1B*-*IL6* T-C-G allele combination was indicated as significant [41]; and no significance was found for the South African subpopulation studied by both Suijkerbuijk et al. [41] and Rahim et al. [42]. However, when all groups were analyzed in the meta-analysis, which increases statistical power, the significance of *IL6* rs1800795 with the GC genotype increasing risk and the protective CC genotype were confirmed.

The rs1800795 polymorphism is located in the promoter region of the *IL6* gene on chromosome 7 at 7p21-p14 and a C to G transition at position -174 is one of the most commonly analyzed polymorphisms [27,28,43]. The polymorphisms in the promoter influence the production of IL-6 in the cells and its release into the blood, and G to C replacement at position -174 results in the attenuation of transcriptional efficacy, and this decreases the production of IL-6 and its concentration in the blood [28,43]. This seems in line with the fact that the CC genotype has been identified as protective in our study. The lack of significance in the South Arican subpopulation [41,42] may have resulted from the fact that this population was not homogenous in either study and represented both contact and non-contact injuries. The other explanation may be related to the fact that several polymorphisms (rs1800795, rs1800796, and rs1800797) exist in the promotor region and the transcriptional regulation is not simply the additive effect of them but complex interactions involving major allele G and even minor variations within nearby SNPs may affect the transcriptional efficacy of rs1800795 [28]. Polymorphisms other than rs1800795 were not examined in the studies [40,41,42] included into meta-analysis in the present study.

IL-6 is pleiotropic, plays a key role in acute response, and also promotes the shift towards either regeneration or a chronic process [13,29,30]. Elevated IL-6 levels have been detected in knees with both acute and chronic damage; however, the patterns of changes were different. It has been reported that IL-6 levels were elevated in patients suffering from osteoarthritis (OA), representing the chronic state, and reached 135.8 ± 224.6 vs. 4.8 ± 0 in healthy controls [44]. Studies on acute knee injuries have demonstrated that IL-6 concentration in synovial fluid increased shortly after injury [21,31] and significantly decreased within 2 weeks after the acute ACL traumatic event [45]. IL-6 levels have also been compared between patients suffering from acute injuries, either ligamentous or meniscal and chronic, end-stage OA patients—being significantly higher in the latter. These findings suggest the role of proinflammatory cytokines in chemical process leading to cartilage damage and the development of OA [46]. In vitro studies have demonstrated that this phenomenon may involve IL-6 stimulation of ACL remnants to the production of perostin, which induces OA-related enzymes such as metalloproteinases, which accelerate cartilage damage [47]. Recently, cytokine profiles have been shown to differ between patients with ACL tears and OA [48]. Although IL-6 was not among the cytokines that differed significantly, its levels were higher in ACL tear patients (285.42 ± 64.36) than OA patients (80.43 ± 56.11) with *p* = 0.051. Therefore, Rai et al. [48] concluded that their data support the previous findings and suggested that the prompt neutralization of IL-6 accumulation in the knee may prevent OA development in the joint with ACL deficiency.

Taking into account the above-mentioned findings, the genetic background responsible for IL-6 production seems critical in both acute and chronic states. The polymorphisms of rs1800795 have been indicated as important, with either the C or G allele being protective, depending on the process. In Egyptian patients, a significant relationship between the rs1800795 C allele and rheumatoid arthritis (RA) has been observed with overexpression of the C allele as either the GC or CC genotype in RA patients [49]. Similar conclusions have been drawn from studies involving Belarusian RA patients, in which the CC genotype occurred with significantly higher frequency [50]. Thus, in a chronic state such as RA, the C allele seems to be associated with increased risk, which contrasts with our results regarding acute injuries, where CC has been identified as a protective genotype. Hall et al. [51] analyzed genetic associations with injury risk in Caucasian male soccer players depending on their maturity status, defined by being pre- or post-peak height velocity. The genetic associations with injuries differed with maturity status and the *IL6* polymorphism was significant only in more mature boys (post-peak height velocity). The prevalence of injuries in general, and also muscle injuries was higher in the CC homozygotes in comparison to the G-allele carriers [51]. This may be interpreted as contrasting with our findings, but it is also likely to be related to the performance of young athletes and the exposure to injury. The Turkish study suggests that the G allele is more prevalent among elite athletes compared to the control group in the Turkish subpopulation, suggesting an association with elite endurance performance. However, the differences in genotype frequencies between elite athletes and the controls were not significant, so more studies are needed to confirm this hypothesis [52].

Studies regarding acute tendon injuries seem to be closer to our results. An investigation of *IL6* rs1800795 SNP in elite Caucasian Spanish male outfield players in relation to musculoskeletal injuries has revealed that the carriers of the GG genotype had a 1.68 times higher risk of hamstring injury in comparison to carriers of the C allele [53]. The authors mentioned Achilles tendinopathy; however, the findings regarding this pathology are inconsistent. The study by Brown et al. [54] on a British subpopulation have shown no significant differences in allele or genotype frequencies between the control group and the patients with Achilles tendinopathy or Achilles tendon rupture. Similarly, a study by September et al. [38] found no independent differences between cytokine gene polymorphisms, including rs1800795, between healthy control subjects and patients with Achilles tendinopathy. However, collectively with the *COL5A1 Bst* UI CC genotype, as a combination of T-T-C-G-A2 and C-T-C-G-AX of COL5A1 BstUI RFLP—IL-1β −31T→C − IL-1β −511C→T − IL-6−172G→C − IL-1RN VNTR were significantly associated with the risk of Achilles tendinopathy.

Cytokine gene polymorphisms have also been investigated as a part of extracellular matrix (ECM) signaling pathways, including *IL6* rs1800795, *IL1B* rs16944, and rs1143627. The interactions between genes encoding collagen proteins, caspases, and cytokines were shown to collectively contribute to the modulation of the overall risk of Achilles tendinopathy [55]. Studies involving the Chinese subpopulation with rotator cuff tears (RCTs) have shown the independent impact of *IL-6* SNP. It was observed that the GG genotype of the rs1800795 polymorphism was associated with a higher risk of RCT and a larger tear size [43] and, in another study, the G allele was also associated with a higher risk of intervertebral disc disease [56].

Another polymorphism examined in this meta-analysis, *IL6R* rs2228145, was not associated with ACLR in the Polish group. However, the CC genotype was proven to be significantly associated with ACLR in the South African subpopulation [40,41,42]. A trend towards significance was also observed for AC genotype overrepresentation in the South African control cohort [42]. The meta-analysis showed no or negligible impact of this SNP on the risk of ACLR and, therefore, did not confirm its role. The pleiotropic IL-6 can signal through soluble forms of IL-6R or via membrane-bound IL-6R, activating intracellular signaling cascades [57]. IL-6, together with its soluble receptor IL-6R, dictates the transition from an acute inflammation to a chronic one, changing the nature of the leucocyte infiltrate [29]. However, in the case of ACLR, the promoter polymorphisms regulating the production of IL-6 seem more important than the polymorphism in the receptor gene.

The association between the *IL1B* rs16944 TT genotype and ACLR was observed in the South African female subpopulation [42], as well as in a haplotype combination of *COL5A1*-*IL1B*-*IL6* T-C-G and *COL5A1*-*IL1B*-*IL6R* T-C-A in Swedish males [41]. No association was found for this polymorphism in the Polish cohort [40]. The meta-analysis may suggest that the CC and CT genotypes increase the risk of ACLR; however, these results are not clear enough and need confirmation. The role of rs16944 in connective tissue pathologies was suggested previously, but the results are not unequivocal. In a study by Brown et al. [54], rs16944 showed no significant association with Achilles tendinopathy; however, the CT genotype was associated with an increased risk of Achilles tendon rupture. September et al. [38] did not show a direct association between rs16944 and the risk of Achilles tendinopathy, but this polymorphism was important for the risk of tendinopathy in the T-T-C-G-A2 and C-T-C-G-AX allele combinations of *COL5A1* BstUI RFLP − *IL1B* rs1143627– *IL1B,* rs16944– *IL6*, and rs1800795 − *IL1RN* VNTR. Another study showed that carriers of the rs16944 CT genotype had a 3.96 times higher risk of Achilles tendon rupture than individuals with the CC or TT genotypes in a British subpopulation [54], which seems to partially confirm the results of this meta-analysis. However, it should be mentioned that the Achilles tendon and ACL differ structurally.

The main limitation of this study is the small number of analyzed articles; however, the total number of participants in the considered studies was 1282, originating from the Polish, South African (Colored and Caucasian), and Swedish subpopulations. Only three studies matched the meta-analysis inclusion criteria, which proves that further research should be conducted concerning the association between genetic variants within genes encoding interleukins and the risk of ACLR. Further research showing the relationship between single-nucleotide polymorphisms within genes encoding interleukins and the risk of soft-tissue (tendon, ligament, and muscle) injury, both independently and in haplotypes, as well as intergenic interactions, should be taken into consideration to give a deeper insight into this problem.

Studies concerning the association between the polymorphisms of genes encoding interleukins and the risk of soft-tissue injury are still scarce. Studying genetic factors as an important contribution to the injury may help to improve injury prevention programs for athletes and for people practicing recreational sports. Personalized programs could be implemented, based on an individual approach, enabling various specialists associated with the athlete to properly prepare them for training sessions and tournaments according to their genetic predispositions and risk factors. Therefore, a comprehensive approach is needed to examine multiple genes in terms of their association with the risk of injury and to use the newest and most innovative methods of statistical analysis. Studies analyzing the association between SNPs and the haplotypes of genes encoding interleukins and the risk of soft-tissue injury should investigate a larger number of polymorphisms and their interactions, also in other subpopulations.

## 5. Conclusions

Anterior cruciate ligament injury is one of the most frequent soft-tissue injuries resulting from physical activity. Based on the findings of this meta-analysis, the polymorphism of the *IL6* rs1800795 GC genotype increased, while CC decreased, the risk of ACLR. Other analyzed polymorphisms were less significant, including no impact of *IL6R* rs2228145 and possible association of *IL1B* rs16944 TT genotype increasing the risk of rupture and the CT genotype having a protective effect. Still, further research is needed to investigate more SNPs within genes encoding interleukins with haplotype and intergenic interaction analyses.

## Figures and Tables

**Table 1:** Characteristics of included studies and their main findings. IL-1—interleukin 1; IL-6—interleukin 6; IL-6R—interleukin 6 receptor; ACLR—anterior cruciate ligament rupture; SNP—single nucleotide polymorphisms; COL5A1—collagen type V alpha 1 chain; CON—control; SA—South African, SWE—Swedish.

Article Details	Il Genes Studied	Case Group	Injury Type	Control Group	Population	Main Results
Lulińska-Kuklik E et al. *Are IL1B, IL6 and IL6R Gene Variants Associated with Anterior Cruciate Ligament Rupture Susceptibility?* J Sports Sci Med. 2019;18(1):137–145.	***IL1B* rs16944** ***IL1B* rs1143627** ***IL6* rs1800795** ***IL6R* rs2228145**	**Polish**: 229 (65 females, 163 males)	**non-contact**: 229	**Polish**: 194 (85 females, 109 males)	**Polish cohort**: Males: Soccer players from the 1st, 2nd, and 3rd divisions of the Polish soccer league, ACLR age: 26 ± 4 years, CON age: 25 ± 3 years **Females**: Soccer players from the 1st division of the Polish soccer league, ACLR age: 25 ± 4 years, CON age: 29 ± 2 years were recruited from sports clubs and wellness centers	***IL6* rs1800795** significantly associated with **ACLR** for the **codominant**, **recessive**, and **overdominant** models (*p* = 0.018 for codominant, *p* = 0.032 for recessive, *p* = 0.007 for overdominant models, *p*-value adjusted for sex) No association between SNP-SNP interaction and ACLR for the codominant, dominant or recessive models No significant differences in the genotype and allele frequencies for *IL6R* rs2228145, *IL1B* rs16944, and rs1143627 (analyzed alone or in haplotype combination) between the ACLR group and the healthy control group
Suijkerbuijk MAM et al. *Functional polymorphisms within the inflammatory pathway regulate expression of extracellular matrix components in a genetic risk dependent model for anterior cruciate ligament injuries.* J Sci Med Sport. 2019;22(11):1219–1225.	***IL1B* rs16944** ***IL6* rs1800795** ***IL6R* rs2228145**	**Swedish**: 79 (36 females, 43 males) **South African**: 98 (17 females, 81 males)	**non-contact**: Swedish: 79, South African: 51 **contact**: South African: 47	**Swedish**: 116 (76 females, 40 males) **South African**: 100 (19 females, 81 males)	**Swedish cohort**: Physically active and unrelated participants recruited via the orthopedic clinics in two major hospitals, age: 19–65 **South African cohort**: Physically active and unrelated participants—ACL group recruited from orthopedic clinic in Cape Town, CON group recruited from sporting clubs and gyms within the greater Cape Town area	For the **South African** cohort, the ***IL6R* rs2228145** A> C **CC** genotype was significantly **overrepresented** (*p* = 0.028) in the **SA-CON** group **compared** to the **SA-ACL** group For the ***COL5A1-IL1B-IL6*** allele combination (in males) the **T–C–G** combination was significantly **underrepresented** (*p* = 0.034) in the **SWE-CON compared** to the **SWE-NON** group, the ***COL5A1-IL1B-IL6R* T–C–A** combination (in males) was significantly underrepresented (*p* = 0.044) in the **SWE-CON** compared to the **SWE-NON** group
Rahim M et al. *Modulators of the extracellular matrix and risk of anterior cruciate ligament ruptures.* J Sci Med Sport. 2017;20(2):152–158.	***IL1B* rs16944** ***IL6* rs1800795** ***IL6R* rs22281457**	**South Africa**: 234 (63 females, 171 males)	**non-contact**: 135 (33 females, 102 males) **contact**: 99	**South Africa**: 232 (102 females, 130 males)	**South Africa**: ACL group recruited from orthopedic clinic in Cape Town, CON group recruited from sporting clubs and gyms within the greater Cape Town area—participants were primarily recreational athletes	When only the **female** participants were analyzed, the ***IL1B* rs16944** genotype **TT** was significantly **underrepresented** in the **CON** group compared to the **NON** subgroup (*p* = 0.039). A trend towards significance (*p* = 0.091) for the **rs2228145** genotype frequencies was noted between the **female CON** group and the **female NON** subgroup, with the **AC** genotype significantly **overrepresented** in the **CON** group (*p* = 0.036).

Abbreviations: IL-1—interleukin 1; IL-6—interleukin 6; IL-6R—interleukin 6 receptor; ACLR—anterior cruciate ligament rupture; SNP—single nucleotide polymorphisms; COL5A1—collagen type V alpha 1 chain; CON—control; SA—South African, SWE—Swedish; CC—cytosine-cytosine; TT—thymine-thymine, AC—adenine-cytosine.

## Data Availability

https://www.crd.york.ac.uk/prospero/#myprospero (accessed on 19 February 2024).
